# Supplementary figures and images for: Prevalence of Viral Frequency-Dependent Infection in Coastal Marine Prokaryotes Revealed Using Monthly Time Series Virome Analysis
Source: mSystems. 2023 Feb 1;8(1):e00931-22. doi: 10.1128/msystems.00931-22 (PMC9948707; doi:10.1128/msystems.00931-22)

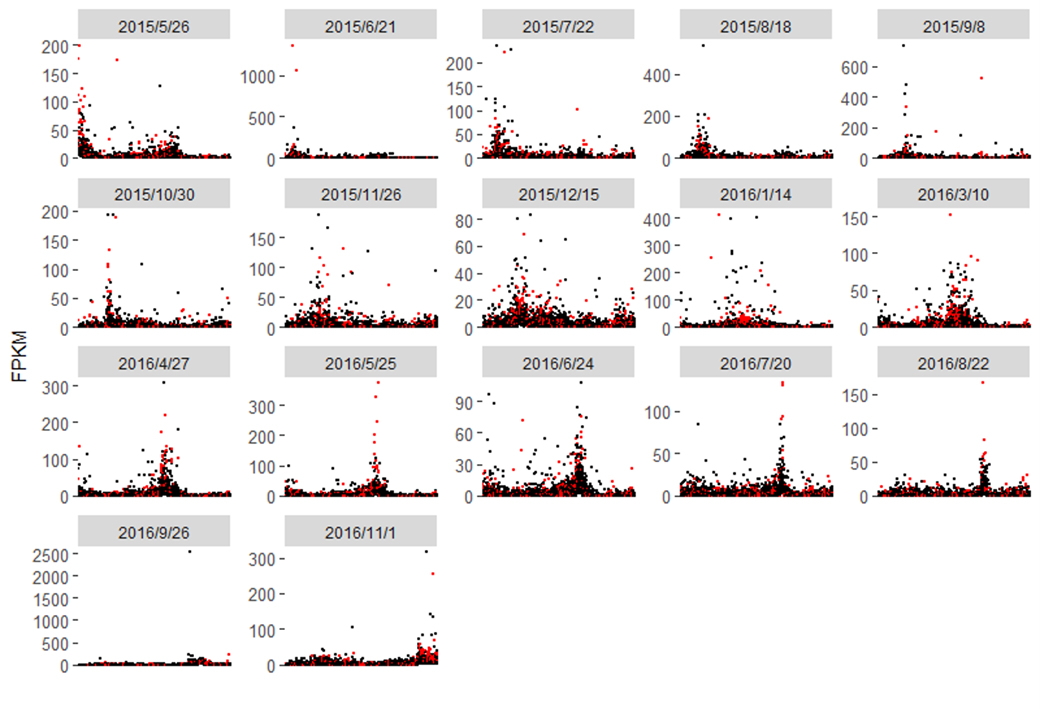

Supplement: FIG S1 [file msystems.00931-22-s0001.png]

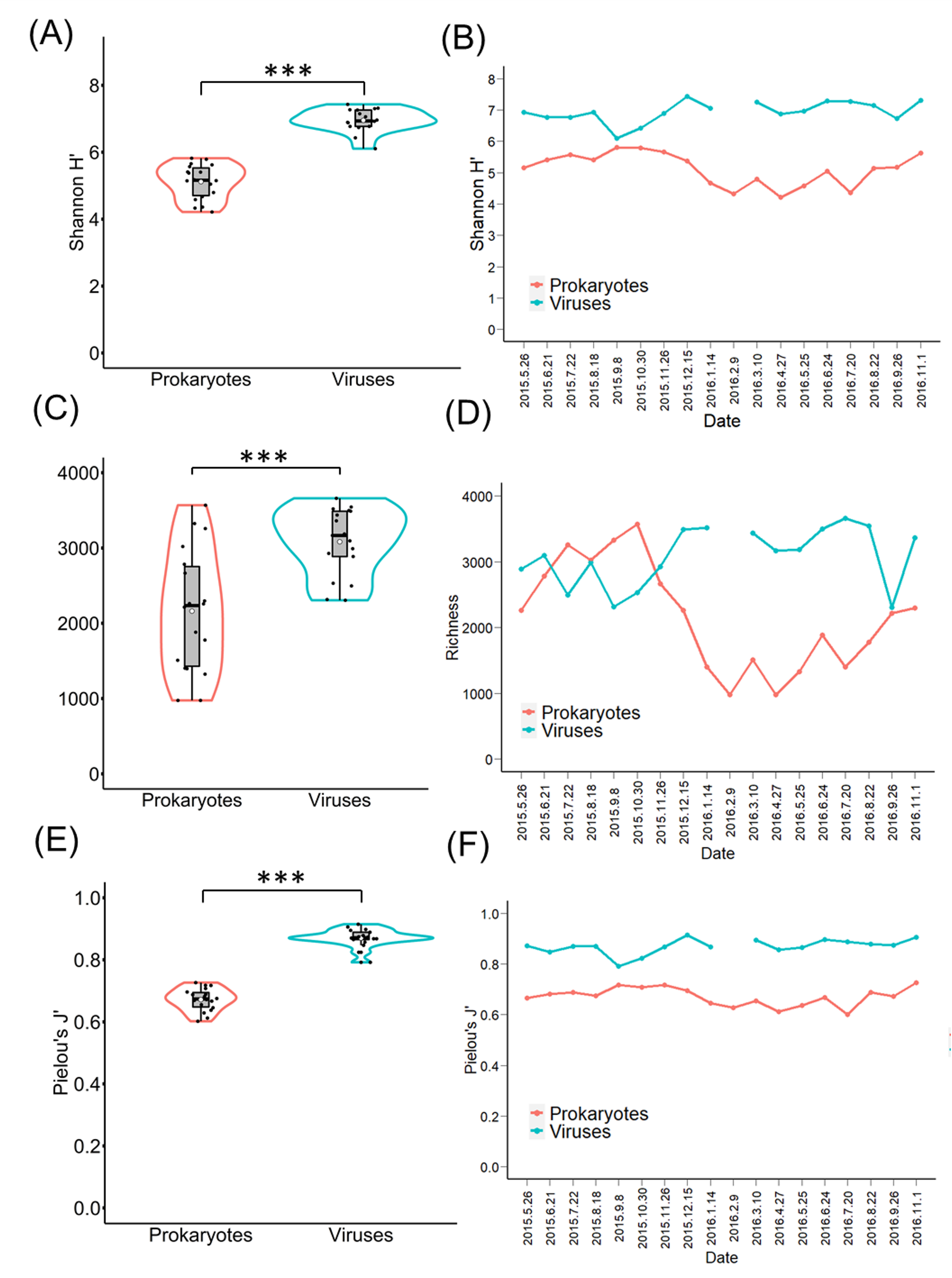

Supplement: FIG S2 [file msystems.00931-22-s0002.png]

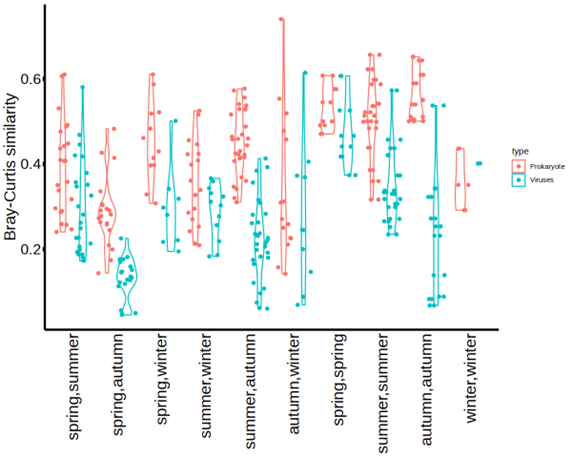

Supplement: FIG S3 [file msystems.00931-22-s0003.png]

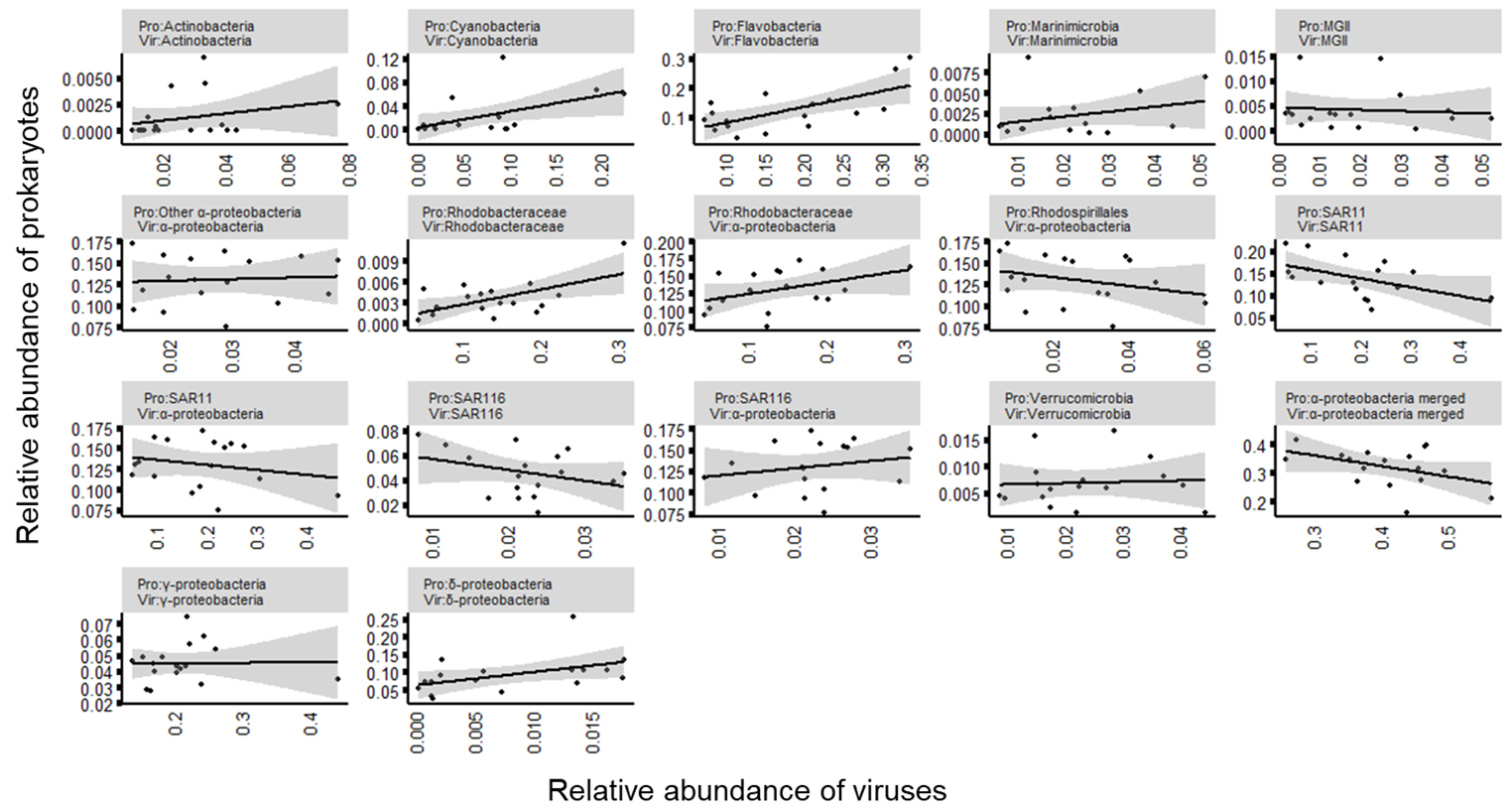

Supplement: FIG S4 [file msystems.00931-22-s0004.png]

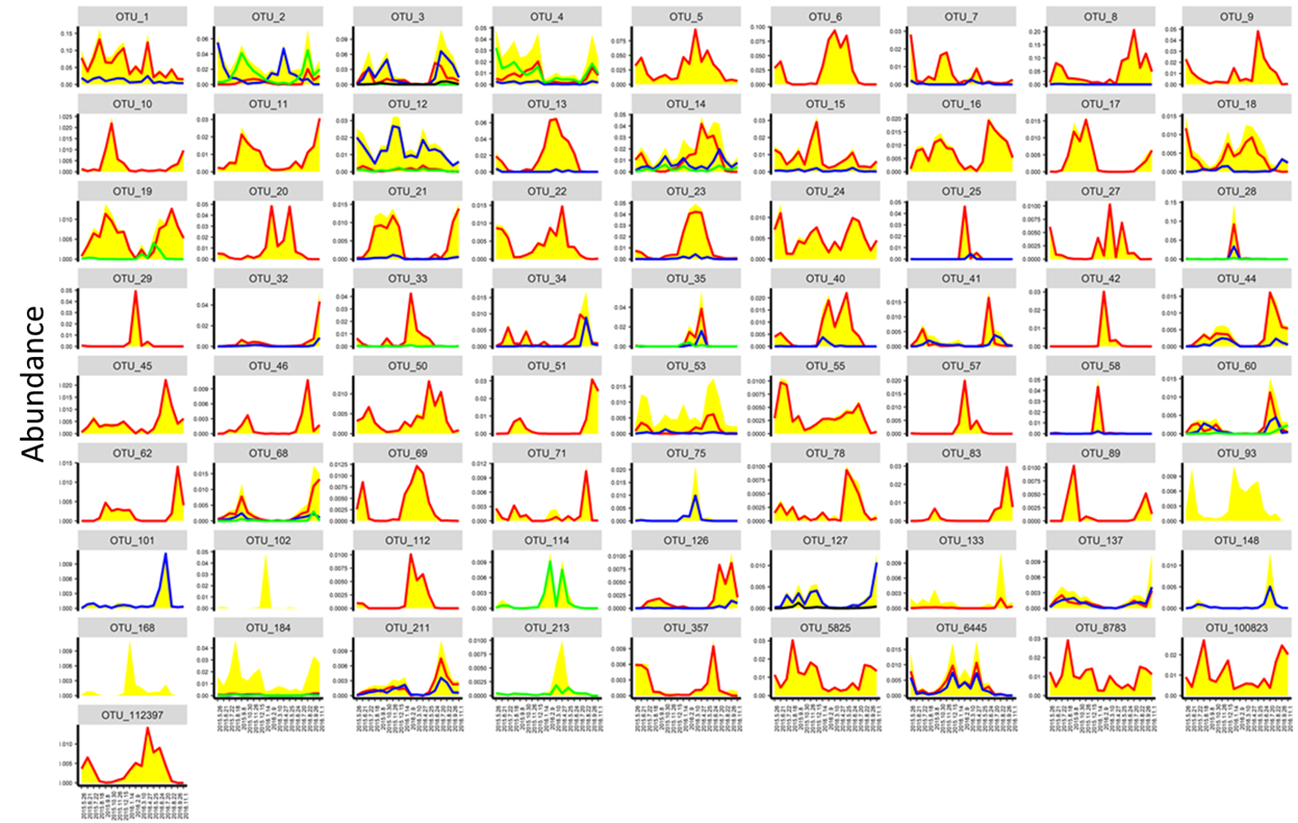

Supplement: FIG S5 [file msystems.00931-22-s0005.png]

(A)

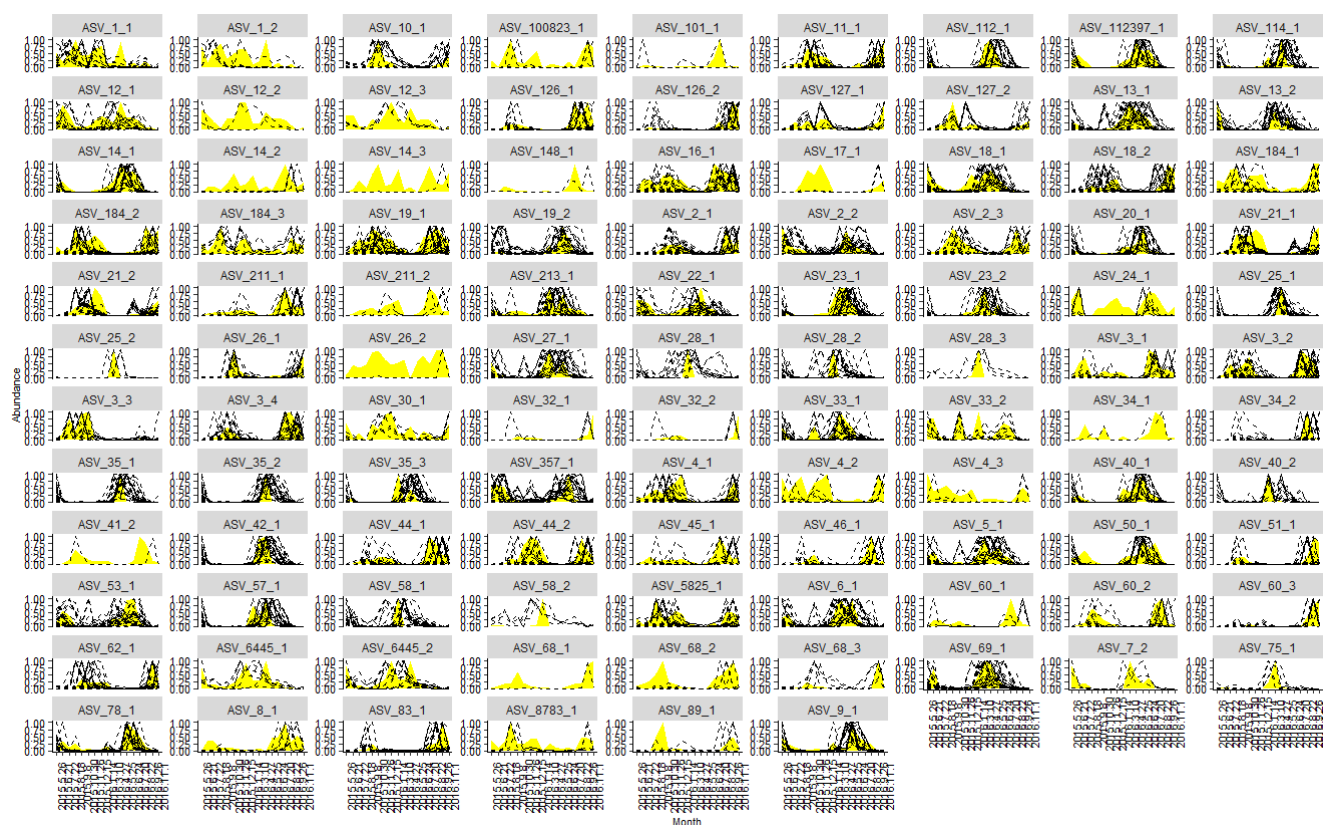

(B)

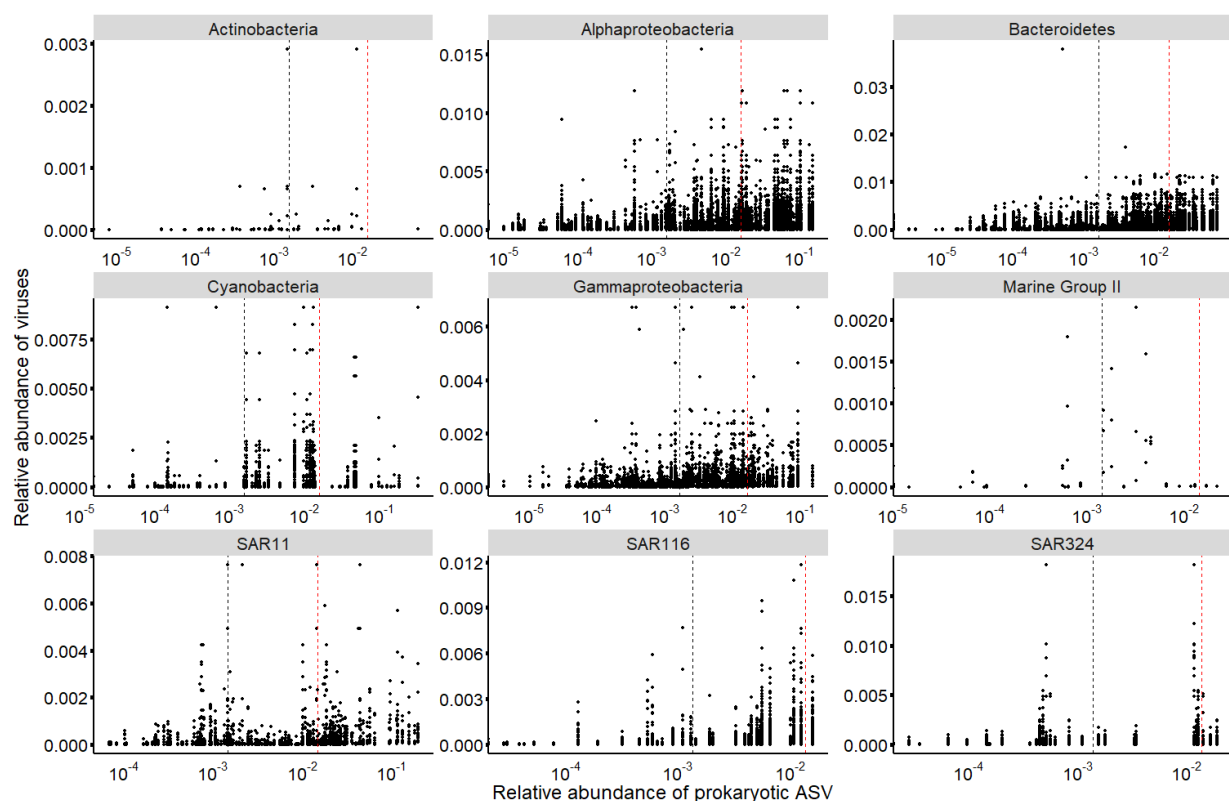

--- 1% ( $\doteq 10^4$  cells)  
---- 0.1% ( $\doteq 10^3$  cells)

(C)

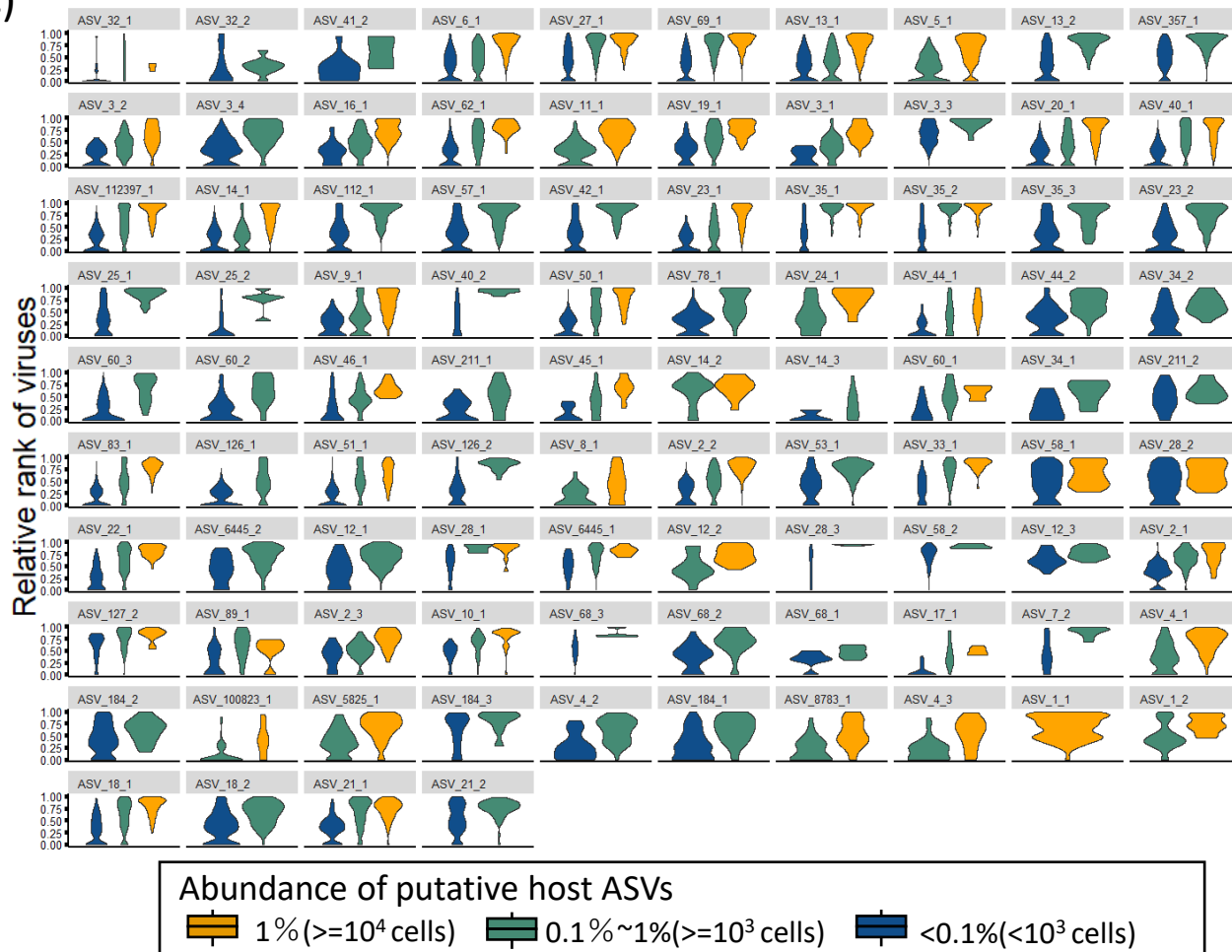

(D)

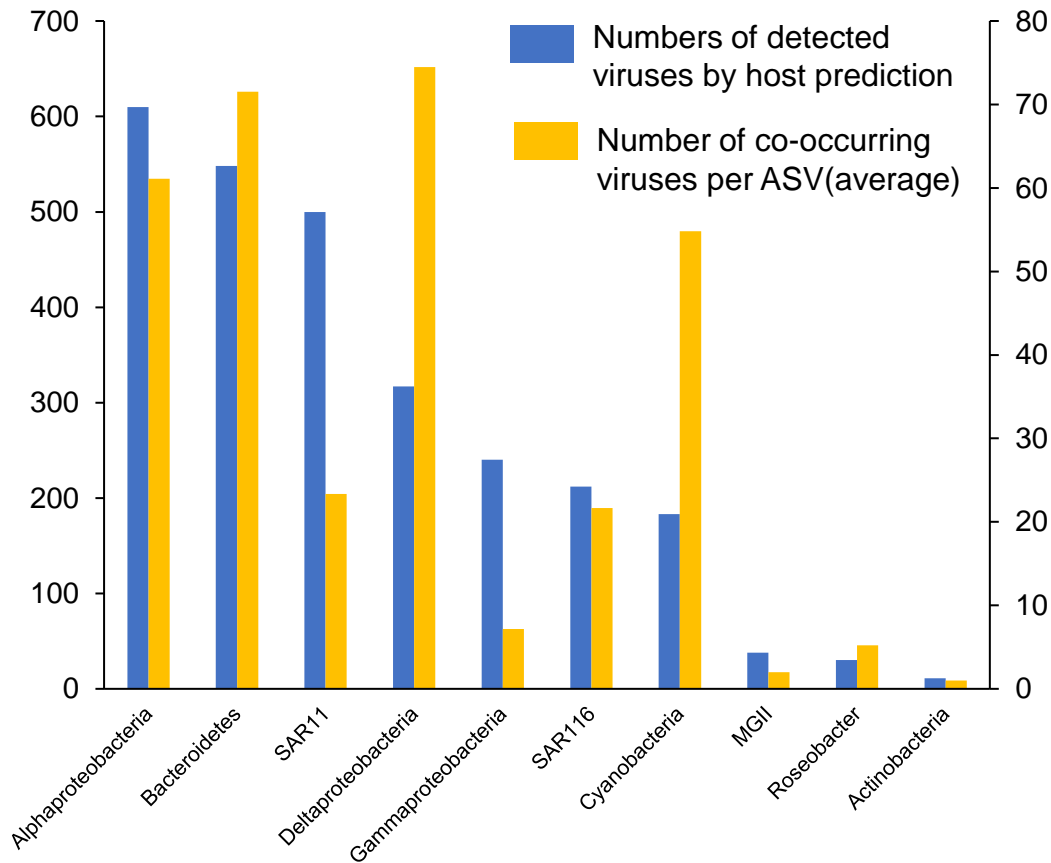

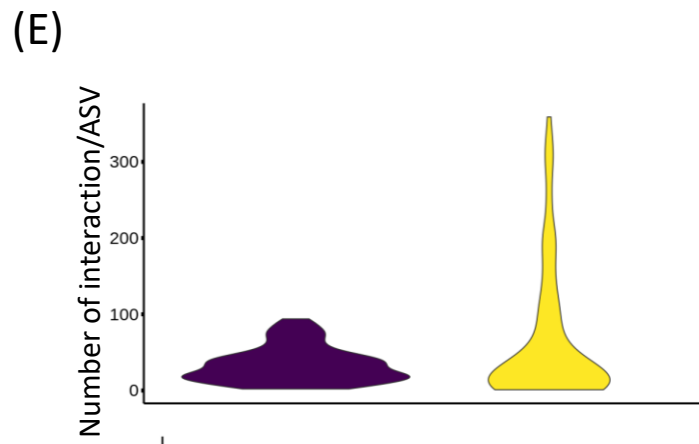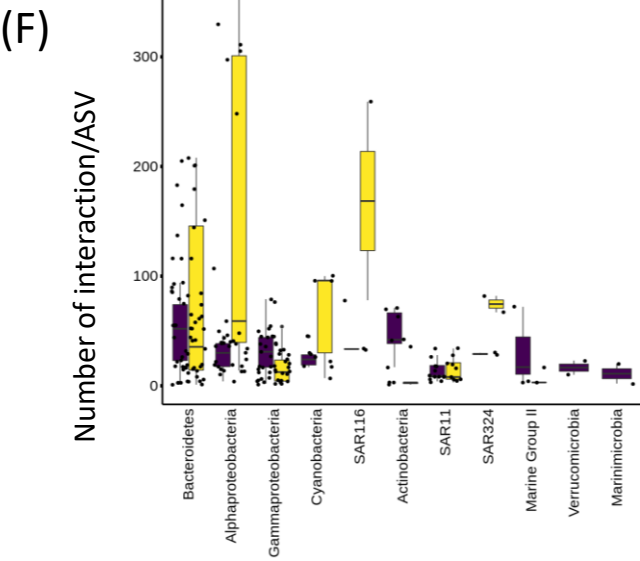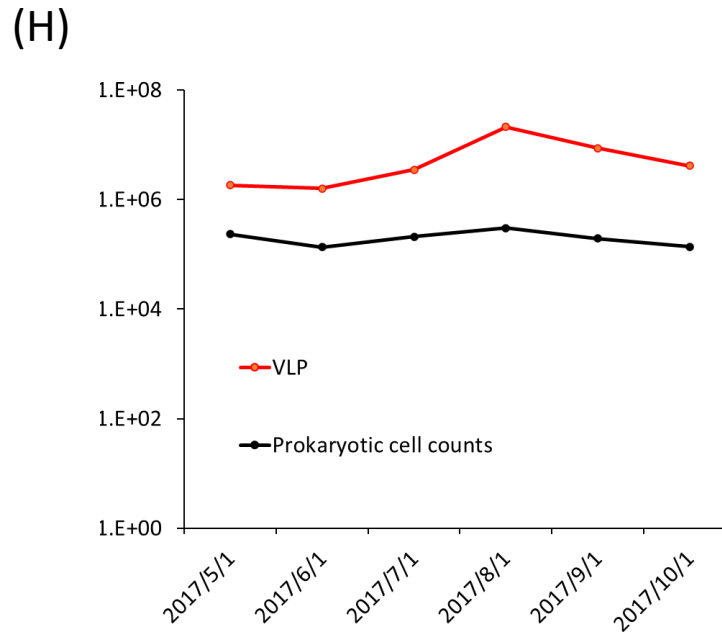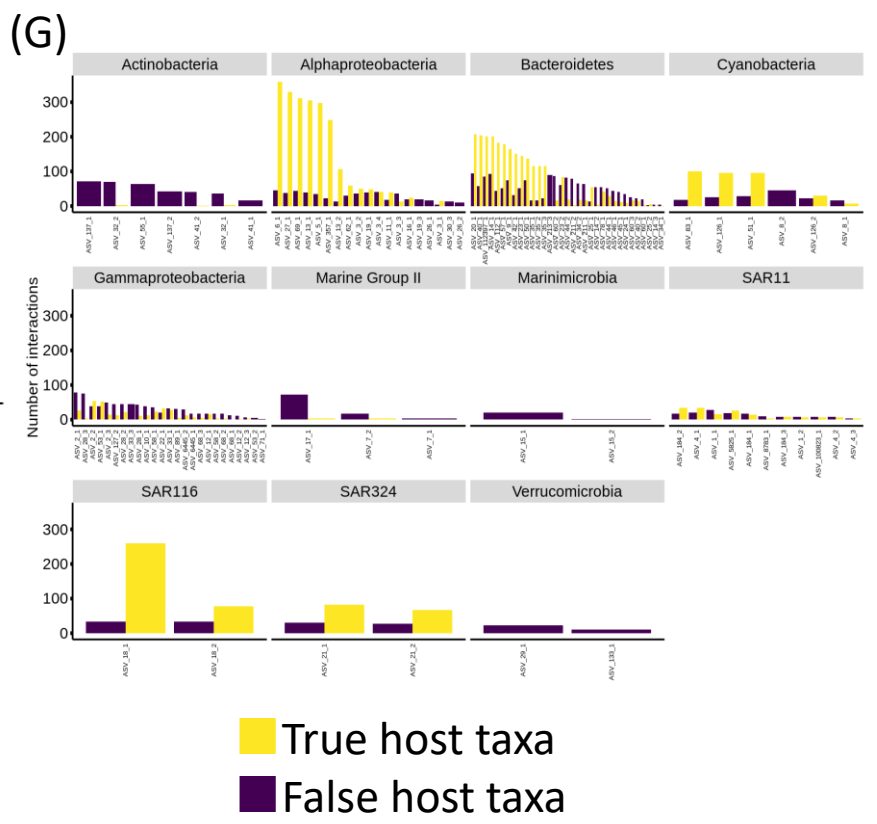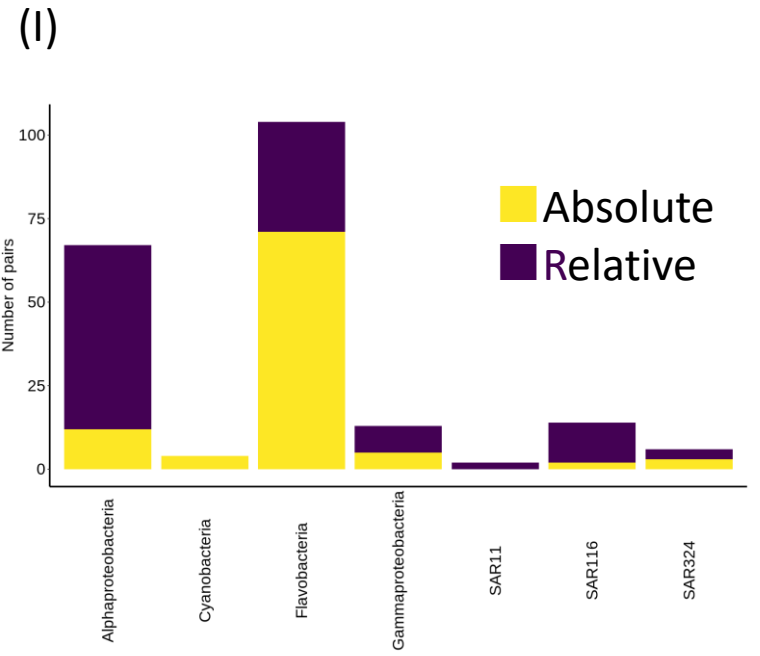

Supplement: FIG S6 [file msystems.00931-22-s0006.pdf]

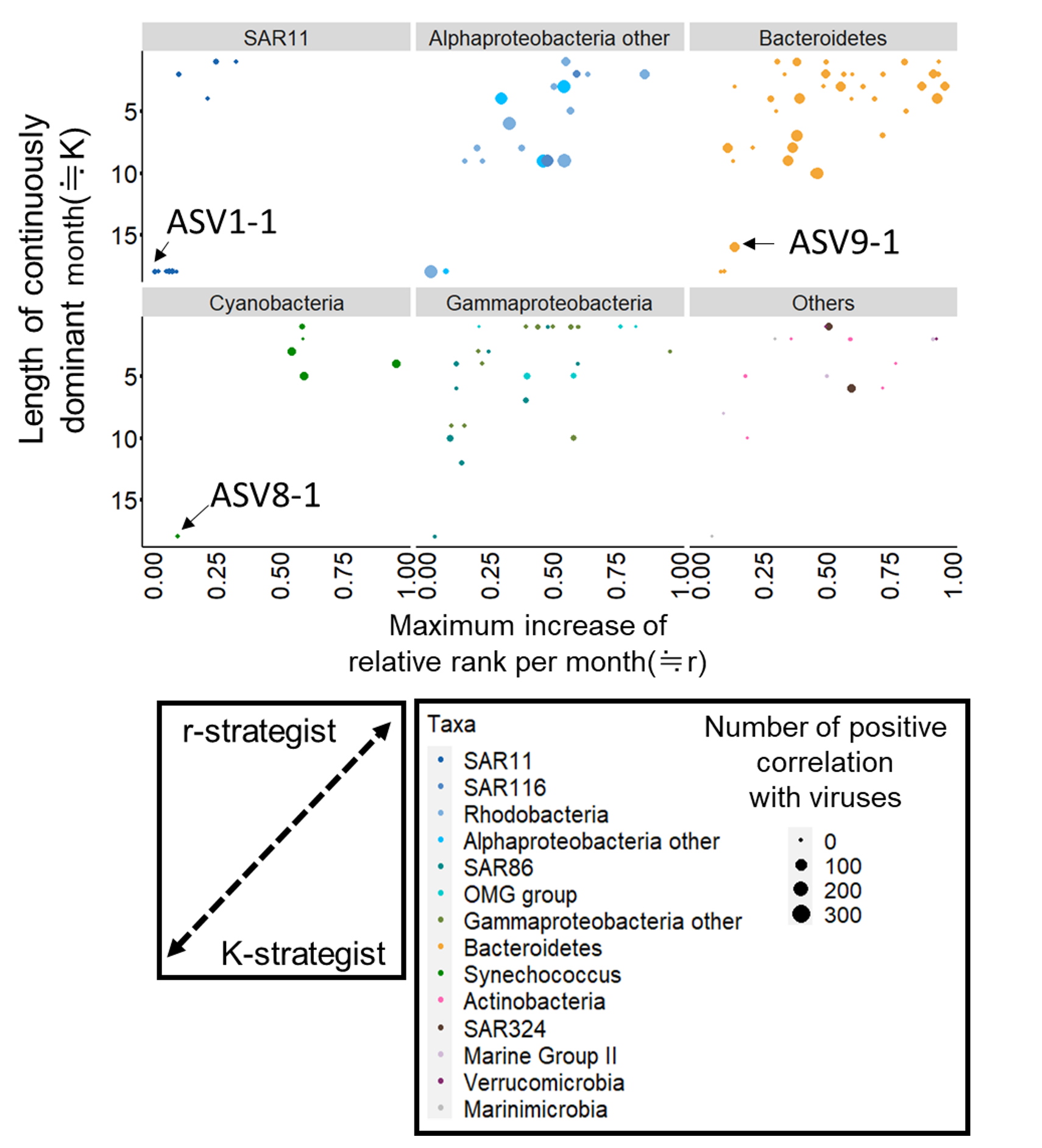

Supplement: FIG S7 [file msystems.00931-22-s0007.png]

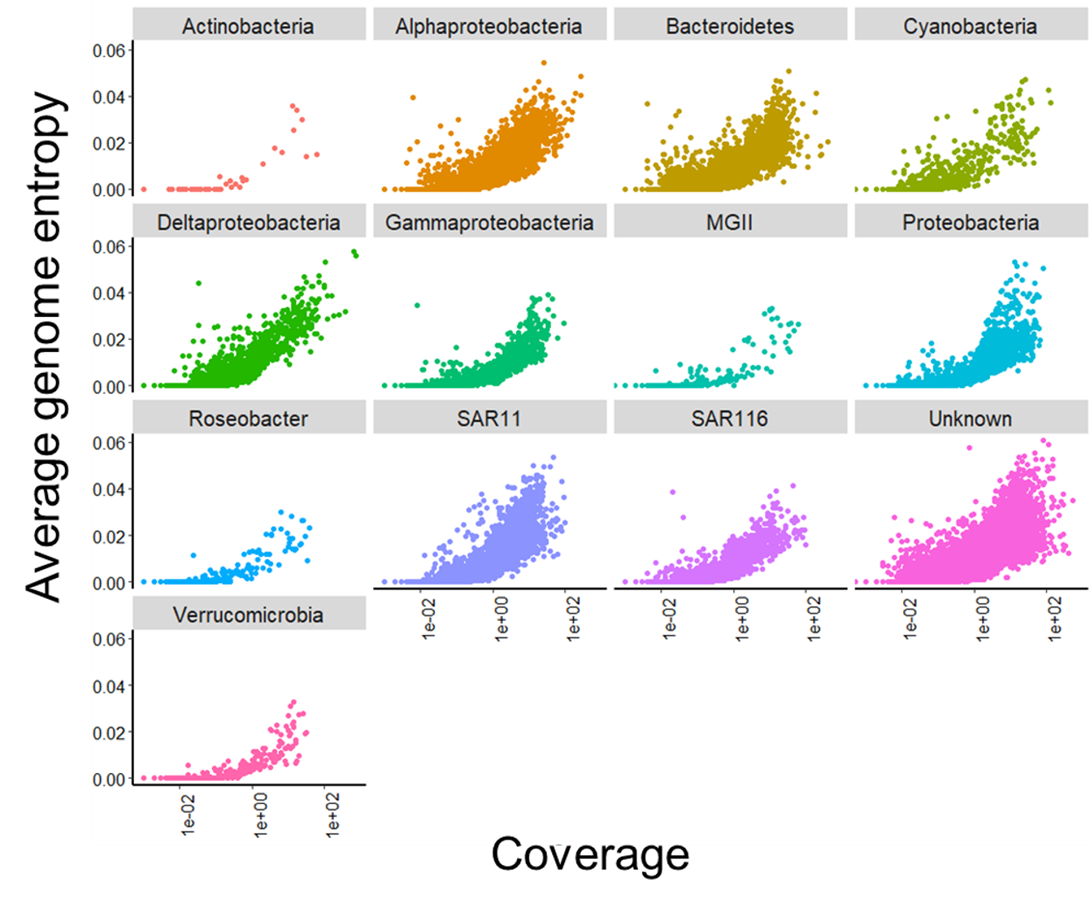

Supplement: FIG S8 [file msystems.00931-22-s0008.png]
